# The Relationship of Cerebrospinal Fluid Biomarkers and Cognitive Performance in Frontotemporal Dementia

**DOI:** 10.21203/rs.3.rs-3945509/v1

**Published:** 2024-02-12

**Authors:** Salih Cayir, Faranak Ebrahimian Sadabad, Adam Mecca, David Matuskey, Arman Fesharaki Zadeh

**Affiliations:** Yale University Radiology and Biomedical Imaging; Yale University Radiology and Biomedical Imaging; Yale University School of Medicine, Alzheimer’s Disease Research Unit; Yale University Radiology and Biomedical Imaging; Yale University School of Medicine, Neurology

**Keywords:** Frontotemporal dementia, CSF, Alzheimer’s Disease, Cognitive decline

## Abstract

**Objective:**

Currently available literature on the relationships between cerebrospinal fluid (CSF) biomarkers and cognitive performance in frontotemporal dementia (FTD) is very limited and inconclusive. In this study, we investigated the association of cognition, as measured with Montreal Cognitive Assessment (MoCA), with CSF levels of total tau (t-tau), phosphorylated tau at threonine 181 (p-tau_181_), and amyloid β 1–42 (Aβ1–42) in a group of patients with FTD and Alzheimer’s disease (AD).

**Methods:**

We conducted a retrospective cohort study with participants selected from the electronic records of patients seen at Yale New Haven Hospital’s Memory Clinic, CT, USA. We included 61 patients, 28 with FTD (mean age = 64.1) and 33 with AD (mean age = 66.8).

**Results:**

T-tau levels negatively and significantly correlated with total MoCA scores as well as the different MoCA index scores in both the FTD (r=−0.469, p < 0.05) and AD (r=−0.545, p < 0.01) groups. There were no significant associations with MoCA scores and p-tau_181_ levels in patients with FTD (r=−0.224, p > 0.05), unlike patients with AD, who exhibited significant correlations (r=−0.549, p < 0.01). Also, Aβ1–42 levels were not significantly correlated with MoCA scores in either of the FTD and AD groups.

**Conclusion:**

CSF concentrations of t-tau are inversely correlated to cognitive performance in patients with FTD and both t-tau and p-tau_181_ in AD. These findings provide valuable insights into the relationship between clinical cognitive performance and tau-related pathology in FTD.

## INTRODUCTION

Frontotemporal dementia (FTD) encompasses a range of clinically and genetically heterogeneous neurodegenerative conditions characterized by varying language and behavior impairments in the early stages of the disease process^1–3^. Behavioral variant FTD (bvFTD) is the most common subtype, encompassing more than half of all FTD cases with primary deficits in executive function and early behavioral alterations, including disinhibition, loss of empathy, hypersexuality, and changes in food preferences^1^. At the same time, semantic variant primary progressive aphasia (svPPA) and non-fluent variant primary progressive aphasia (nfvPPA) are in the language spectrum of FTD and present with a progressive decline in language abilities over the disease progression^2^. More specifically, svPPA typically involves prominent anomia, surface dyslexia, and semantic language dysfunction, often leading to circumlocution, whereas nfPPA involves problems with language production and grammatical errors^2^. Some patients may have clinical features of both FTD and motor neuron disease (MND), leading to a syndromic diagnosis of FTD-MND^4^. Also, in terms of underlying pathology, tau protein inclusions in the absence of amyloid β deposits are a hallmark of FTD-related tauopathies (i.e., corticobasal degeneration (CBD), progressive supranuclear palsy (PSP)), a subset of FTD neuropathology^5, 6^.

Disease-related cerebrospinal fluid (CSF) and/or blood biomarker alterations have been shown in many neurodegenerative conditions such as stroke, traumatic brain injury, Parkinson’s disease, and different dementias^7–9^. Moreover, CSF analyses of total tau (t-tau), phosphorylated tau at threonine 181 (p-tau_181_), amyloid β 1–42 (Aβ1–42) levels, and p-tau_181_/ Aβ1–42 ratio are of high importance as highly sensitive and specific biomarkers for identifying Alzheimer’s Disease (AD) pathology and guiding diagnostic and treatment decisions in clinical practice^10^. There is also a growing interest in using these biomarkers to assess the disease severity and predict the progression of FTD^11^. However, CSF biomarkers are not routinely ordered in clinical practice and are mainly used by clinicians for differential diagnostic methods in cases without a clear etiology in FTD^12, 13^.

The literature connecting CSF biomarkers and cognitive performance in FTD is sparse. Very few studies have specifically examined FTD, with pertinent data sometimes incorporated into reports on other neurological disorders. In these limited studies, the authors did not find a significant association between CSF biomarkers and cognition as measured with mini-mental state examination (MMSE) in patients with FTD^12, 14–16^. Notably, none of these studies utilized the Montreal Cognitive Assessment (MoCA) to measure cognitive performance, a brief screening tool with enhanced sensitivity to frontal lobe dysfunction compared to other commonly used cognitive screens like MMSE^17^. MoCA has a total of six domains with a maximum of 30 points: (1) memory; (2) executive function; (3) attention; (4) language; (5) visuospatial function; and (6) orientation^18^. Recently a novel index scoring system for each domain of MoCA was developed by Julayanont et al., which yields domain-specific index scores that allow for assessing the different aspects of cognitive performance^19^.

Taken together, currently available literature on the relationships between CSF biomarkers and cognitive performance in FTD is very limited. Also, there is a need in the literature for studies to examine cognition in FTD with cognitive measures more specific to FTD, such as MoCA. Thus, the primary objective of this retrospective cross-sectional study is to explore potential correlations between CSF t-tau, p-tau_181_, and amyloid β 1–42 levels and cognitive performance assessed using MoCA in patients with FTD. Moreover, to increase the study’s validity and as a comparison to FTD, we included data from a group of patients with AD in all analyses.

## METHODS

### Study Design and Participants

We conducted a retrospective cross-sectional study with participants selected from the electronic records of patients seen at Yale New Haven Hospital’s (YNHH) Memory Clinic, CT, USA, covering the period from 2015 to 2023. All data were retrieved from electronic medical files. The inclusion criteria involved patients who met the following conditions: (1) inpatient or outpatient visit for evaluation by a cognitive disorders specialist at YNHH Memory Clinic between 2015 and 2023 with minimum of two years of follow-up; (2) adults with a clinical diagnosis of probable FTD or AD by an expert multidisciplinary team on neurodegenerative diseases using international consensus criteria^1, 2, 20^; (2) has completed at least one lumbar puncture (LP) with available CSF t-tau, p-tau_181_, and amyloid β 1–42 levels in the chart; (3) having an available MoCA score within six months of their LP date. Patients with a history of chronic substance use or severe psychiatric illnesses, that could affect cognitive functioning, such as alcohol use disorder or severe depression were excluded. Patients with evidence of a large cerebral mass, infarction, and/or hemorrhage in their available neuroimaging or a history of severe cerebrovascular disease were excluded. Lastly, we did not include the patients with CBD and/or PSP in our study.

Chart review resulted in 28 FTD and 33 AD patients meeting the inclusion and exclusion criteria. The patients were further classified according to subtypes as bvFTD (n = 17), svPPA (n = 5), nfvPPA (n = 4), and FTD-MND (n = 2)^1, 2, 4^. To increase power in the correlation analyses, all FTD subtypes were combined, but data for each subgroup were reported in the supplementary material (Table S1A and S1B).

### Demographic and Clinical Variables

MoCA scores, CSF results, and demographic information, including age, sex, years of education, and months since disease onset, were extracted from each patient’s chart. Our database includes item-level data for MoCA scores; thereby, we calculated the index scores (memory, executive function, visuospatial function, language, attention, orientation) based on the validated methods reported previously^18, 19^. LP procedures were carried out at YNHH by neurology residents or attending neurologists for each patient. Subsequently, all collected CSF samples were dispatched to commercial laboratories (Athena Diagnostics or Mayo Clinic), where an ADmark^®^ ELISA kit (available at https://www.athenadiagnostics.com/view-full-catalog/a/admark-reg;-alzheimer-s-evaluation and https://www.mayocliniclabs.com/test-catalog/overview/607273) was employed for comprehensive CSF analysis. This analysis encompassed the quantification of CSF concentrations of Aβ1–42, t-tau, and p-tau_181_, with the p-tau_181_ /Aβ1–42 ratio being calculated as well.

### Statistical Analysis

The patient groups were characterized using mean and standard deviation values for continuous variables. Aβ1–42, tau, and p-tau_181_ were expressed in nanograms per milliliter. After the normality of variables was assessed using the Kolmogorov–Smirnov test (p > 0.05), unpaired t-tests (significance level alpha = 0.05) were used to examine differences in continuous variables between FTD and AD groups, and the chi-square test (alpha = 0.05) was employed for categorical variables. Relationships between CSF (t tau, p-tau_181_ and Aβ1–42 ) and MoCA scores were assessed using Pearson’s correlation (two-tailed, alpha = 0.05). P-values were not corrected for multiple comparisons, considering the exploratory nature of the study. Statistical analysis was performed with IBM SPSS (v. 29.0) and GraphPad Prism (v. 9.0).

## RESULTS

### Demographic and Clinical Characteristics of Patients

A total of 61 patients were included in this study—28 with FTD and 33 with AD. Demographic data for diagnostic groups are presented in Table 1. There was significant difference in sex distribution among FTD and AD (p < 0.05). Detailed data for demographic information and MoCA index scores across different FTD sub-diagnoses are provided in the supplementary material (Table S1A).

### Correlations between CSF Biomarkers and Clinical Characteristics of Patients

**Figure 1:**
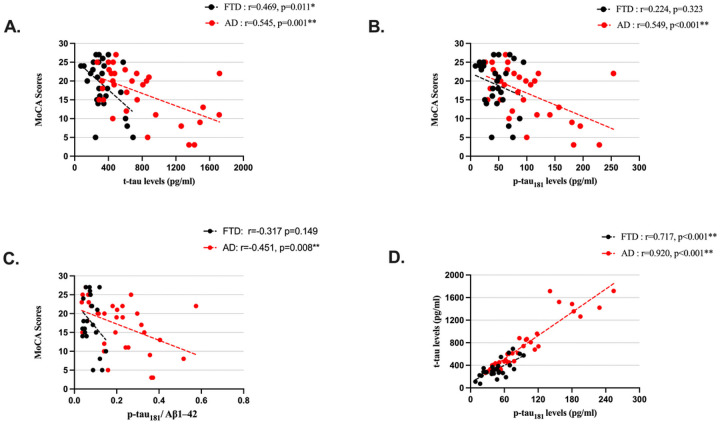
Scatterplots of the associations between t-tau, p-tau_181_ levels, and Montreal Cognitive Assessment (MoCA) total scores. p-tau_181_: Phosphorylated Tau at Threonine 181; t-tau: Total Tau. *: Correlations are significant at 0.05 level. **: Correlations are significant at 0.01 level.

Figure 1 illustrates the correlations between MoCA scores with t-tau and p-tau_181_ levels. There were significant negative associations between t-tau levels and MoCA scores in both FTD and AD groups, with a stronger correlation observed in the AD group (r=−0.469, p = 0.011; r=−0.545, p = 0.001, respectively). Significant negative associations between p-tau_181_ levels and MoCA scores were observed in the AD group (r=−0.549, p < 0.001) (Fig. 1); there were no significant correlations between p-tau_181_ levels and MoCA scores in the FTD group (r=−0.224, p = 0.323) (Fig. 1). There was no significant correlations between Aβ1–42 levels and MoCA scores in either group (r = 0.259, p = 0.245; r = 0.232, p = 0.194; FTD and AD, respectively). However, the p-tau_181_ /Aβ1–42 ratio and MoCA scores were significantly negatively correlated in patients with AD but not in the patients with FTD (r=−0.451, p = 0.008; r=−0.317, p = 0.149, respectively) (Fig. 1). T-tau and p-tau_181_ levels were significantly correlated in both groups (r = 0.717, p < 0.001; r = 0.920, p < 0.001; FTD and AD, respectively) (Fig. 1). There was no significant correlation between Aβ1–42 and t-tau (r=−0.161, p = 0.423; r=−0.140, p = 0.436; FTD and AD, respectively) or p-tau_181_ (r=−0.381, p = 0.061; r=−0.206, p = 0.251; FTD and AD, respectively).

In exploratory analyses, when CSF biomarkers were correlated with individual cognitive domains of MoCA, there were negative significant associations between t-tau levels and executive, attention, language, visuospatial, and orientation functions in patients with FTD (p < 0.05), but no significant associations were observed with memory function (p > 0.05) (Table S2).

### Group Differences in CSF Biomarkers

Table 2 summarizes CSF levels of t-tau, p-tau_181_, Aβ1–42, and p-tau_181_ /Aβ1–42 ratios. Compared to patients with AD, t-tau (330.9 vs. 766.6 pg/ml, p < 0.01) and p-tau_181_ (46.07 vs. 96.8 pg/ml, p < 0.01) levels were significantly lower in patients with FTD (Table 2). Aβ1–42 levels were significantly higher in patients with FTD compared to AD (707.7 vs. 543.2 pg/ml, p < 0.05) (Table 2). The p-tau_181_ /Aβ1–42 ratio was significantly higher in patients with AD compared to FTD (0.07 vs. 0.21, p < 0.01) (Table 2). Detailed data for CSF biomarkers across different FTD disease subtypes are provided in the supplementary material (Table S1B). No significant differences were found among FTD groups (p > 0.05) (Table S1B).

### Discussion

This study investigates the relationship between conventional CSF biomarkers—Aβ1–42, t-tau, and ptau_181_ — and MoCA scores in FTD and AD cohorts. According to these findings, t-tau levels inversely correlate with MoCA scores in both FTD and AD groups, with a more pronounced association in the AD group. In contrast, there were no associations between MoCA scores and p-tau_181_ levels in patients with FTD, whereas patients with AD exhibited significant correlations. Also, our results suggest lower t-tau and p-tau_181_ levels, as well as a reduced p-tau_181_/Aβ1–42 ratio and elevated Aβ1–42 levels in patients with FTD compared to those with AD.

The analysis of CSF biomarkers revealed a negative trend of t-tau levels when correlated with MoCA scores in patients with FTD. Furthermore, when exploring the relationship between t-tau levels and different MoCA index scores in patients with FTD, we observed negative correlations across executive, visuospatial, language, attention, and orientation, but not memory domains. Notably, the lack of significant results in the memory domain may be attributed to the relatively preserved memory function in the early stages of FTD (our cohort had a disease duration of 27±20 months)^21^. This is consistent with a recent study conducted by Ang et al., which showed relatively low validity of the MoCA memory domain compared to executive and language domains in a large cohort (602 subjects) of patients with FTD^22^.

These findings contradict the limited literature on CSF biomarkers and cognition in FTD. Casoli et al. examined CSF biomarkers and cognitive status, as measured by MMSE, in twenty-one patients with FTD and found no significant associations^15^. Skillback et al. conducted a similar study with MMSE as the cognitive measure in a larger cohort but did not find significant correlations^16^. Two other studies with similar designs and measures reported similar results^12, 14^. The discrepancy between the previous literature and our study results might be explained by a different cognitive measure utilized in our study as the limited effectiveness of the MMSE as a screening tool for individuals with FTD has been previously proposed^23^. This limitation is attributed to the MMSE’s primary emphasis on cognitive domains that are mainly affected in AD, with insufficient attention to executive function, a critical aspect in the comprehensive assessment of patients with FTD^17, 24^. In contrast, the MoCA we used in the current study includes specific items that assess frontal lobe processes, enhancing its sensitivity to detect frontal lobe dysfunction compared to the MMSE^18, 19^. Taken together, future studies with more robust clinical cognitive measures sensitive to FTD pathology might be essential to have a better picture of potential biomarkers.

There was no significant correlation between p-tau_181_ levels and MoCA scores in patients with FTD. This is an expected finding since the p-tau_181_ is considered an AD-specific marker, and several previous studies have reported that CSF p-tau_181_ does not play a discriminative role in distinguishing between FTD and healthy controls^25, 26^. When examining the correlations between t-tau and p-tau_181_ levels and MoCA scores in patients with AD, we found a significant correlation for both biomarkers with a similar magnitude. This might be an important finding since there is no consensus related to the association between CSF biomarkers and cognitive function in AD so far^27^.

In both FTD and AD patient groups, no significant correlation was observed between the concentrations of Aβ1–42 and MoCA scores. This absence of correlation may not be surprising in patients with FTD since these conditions typically involve minimal or no amyloid pathology in the brain^5^. On the other hand, our findings in AD patients align with prior research, indicating a stronger correlation between cognitive measures and tau markers when compared to Aβ1–42 in AD^28, 29^. In a recent study by Ramanan et al. 2023 involving a large cohort of patients with AD, no association was found between the Aβ42/40 ratio and MMSE scores in the overall sample, whereas p-tau_181_ levels exhibited a positive association with MMSE scores^30^. The lack of significant findings in our study and previous studies related to Aβ1–42 levels and cognition in patients with AD can be attributed to a hypothesis proposing that amyloid load remains relatively stable in the brain throughout various clinical phases of AD and does not exhibit substantial changes as the disease progresses to more severe stages^31^. Furthermore, our data revealed only a weak correlation between tau levels and Aβ1–42 in both diagnostic groups, indicating a limited or absent direct relationship between Aβ1–42 plaque load and tau-related pathology. This observation lends support to the idea that while Aβ1–42 accumulation in the brain may play a crucial role in initiating disease pathology in AD, it may not be the primary driving force behind neurodegeneration and subsequent cognitive decline in the later stages of the disease^31, 32^.

Consistent with the literature, we found lower t-tau and p-tau_181_ levels, higher Aβ1–42 levels, and a decreased p-tau_181_/Aβ1–42 ratio in patients with FTD. It is not unexpected to see an increased p-tau_181_ level and decreased Aβ1–42 levels in patients with AD since the phosphorylation of the tau protein at the threonine 181 epitope and the amyloid plaque load in the brain suggest a process unique to AD and not commonly observed in other non-AD dementias^25^.

On the other hand, t-tau is a non-specific marker for axonal and neuronal degeneration, and elevated t-tau concentrations may be observed in various forms of non-AD dementias^33^. Given that axonal degeneration occurs as part of any neurodegenerative process, one would expect to see a comparable t-tau level with AD in CSF of patients with FTD. However, this is not the case in our study or previous literature, as the increase in tau concentrations in AD clearly exceeds the increase in FTD, as shown in different meta-analyses and large cohort studies^16, 34–36^. It is still not well known why CSF t-tau levels remain relatively low in FTD compared to AD. However, there are a few possible explanations for this phenomenon.

First, the variability may stem from the inclusion of different subgroups of patients with FTD in various studies, including the current study. These subgroups might associate with distinct tau levels, potentially mirroring the diverse spectrum of tau concentrations observed in postmortem examinations of FTD patients’ brains^12, 37^. Furthermore, even in the same group of patients, different tau profiles could be caused by different genetic and pathological backgrounds, such as FTD-tau, FTD-TDP, and, more recently, FTD-FUS^5, 6^. An alternative explanation could be that tau pathology observed in FTD might involve disease-specific tau fragments that undergo truncation in a manner not detected by current assays and escape the current ELISA method, which specifically targets only the mid regions of the tau protein^7^. Lastly, the hypothesis is that the sequestration of tau proteins in the brain after neuronal death in the form of Pick bodies or balloon cells might prevent the leakage of the tau proteins into the CSF^38^. Some evidence supporting this hypothesis arises from relatively low levels of CSF tau found in patients with CBD, a clinical phenotype of FTD, which is associated with balloon cells in the brain that contain high levels of tau^39^.

Our study does have several limitations. First, we didn’t have pathological confirmation of diagnoses for our study patients. However, we sought to minimize this bias by ensuring the highest clinical accuracy, including patients with at least two years of clinical follow-up. Second, we don’t have reported genetic results for the patients we included in this study. Considering the pathologically and genetically heterogeneous background of FTD, it might be important to group patients based on their genetic results. Also, patients with FTD and AD were not balanced for biological sex, which might introduce a sex-dependent bias given the suggested literature for more robust changes in tau levels with female patients with AD^40^. However, when we examined the sex differences in CSF biomarkers in both groups, there was no significant difference (data not shown). Additionally, using MoCA as the sole measure of cognition may have limited accuracy compared to more comprehensive cognitive assessments. Another limitation stems from the relatively small sample sizes, which could lead to non-significant results in some analyses. Furthermore, our CSF data originated from two different centers, which might introduce some variability in the results. However, it’s worth noting that there were no significant differences in CSF biomarkers when comparing different centers within individual diagnosis groups (data not shown).

### Conclusions

Regardless of the clinical heterogeneity of our study sample, we report that CSF concentrations of t-tau are inversely correlated to cognitive performance in patients with FTD and both t-tau and p-tau_181_ in AD. Our results indicate that high CSF t-tau concentrations may reflect worse cognitive impairment in patients with FTD. These findings provide valuable insights into the relationship between clinical cognitive performance and tau-related pathology in FTD and may inform the design of future research focused on cognition and biomarkers in FTD.

## Figures and Tables

**Table 1:** Demographic characteristics of participants, by diagnosis group

	FTD (n = 28)		AD (n = 33)		p values
	M	SD	M	SD	
**Age (mean)**	64.1	8.9	66.8	8.8	0.245
**Sex (male/female)**	21/7		13/20		0.005**
**Education (years)**	14.3	3.4	14.7	3.01	0.608
**Disease Duration (months)**	27	20	26.8	17.6	0.970
**MoCA**	19.04	6.5	17	6.6	0.279
**Time between LP and MoCA (months)**	2.8	1.8	2.7	1.7	0.818

The values are given as the means ± standard deviation (SD). Abbreviations: MoCA-TS; Montreal Cognitive Assessment; LP: Lumbar puncture. Statistical Significance Between FTD and AD was tested using independent samples t-test and Pearson Chi-Square test.

**: Results were significant at 0.01 level.

**Table 2:** Cerebrospinal fluid (CSF) Levels of t-tau, p-tau181, Aβ1–42 and p-tau181/ Aβ1–42 ratios of participants, by diagnosis group

	FTD (n = 28)		AD (n = 33)		p values
	M	SD	M	SD	
**t-tau (pg/ml)**	330.9	153.07	766.6	434.3	< 0.001**
**p-tau**_**181**_ **(pg/ml)**	46.07	22.1	96.8	58.1	< 0.001**
**Aβ1–42**	707.7	225.8	543.2	245.4	0.015*
**p-tau**_**181**_ **/Aβ1–42**	0.07	0.03	0.21	0.13	< 0.001**

The values are given as the means ± standard deviation (SD). The unit of measure for CSF markers is pg/ml. Abbreviations: p-tau_181_; Phosphorylated Tau at Threonine 181, t-tau; Total Tau. Statistical Significance Between FTD and AD was tested using an independent samples t-test.

*: Results are significant at 0.05 level.

**: Results are significant at 0.01 level.

## Data Availability

The datasets used and/or analyzed during the current study are available from the corresponding author on reasonable request.
